# Short- to mid-term outcomes of refractory electrical storm patients listed for urgent heart transplantation

**DOI:** 10.1016/j.jhlto.2026.100529

**Published:** 2026-02-28

**Authors:** Miloud Cherbi, Philippe Maury, Pierre Groussin, Mathilde Hamel-Bougault, Francis Bessière, Matteo Pozzi, Fabrice Extramiana, Charles Guenancia, Audrey Sagnard, Sandro Ninni, Céline Goemine, Pascal Defaye, Aude Boignard, Baptiste Maille, Vlad Gariboldi, Pierre Baudinaud, Anne-Céline Martin, Laure Champ-Rigot, Katrien Blanchart, Jean-Marc Sellal, Christian De Chillou, Laurence Jesel-Morel, Michel Kindo, Corentin Chaumont, Frédéric Anselme, Marine Arnaud, Erwan Flecher, Léa Benabou, Maxime Faure, Shaida Varnous, Estelle Gandjbakhch, Paul Gautier, Redwane Rakza, Karim Benali, Raphael P. Martins, Clément Delmas

**Affiliations:** a Electrophysiology Department, University Hospital Toulouse, Toulouse, France; b Université Paul Sabatier - Toulouse III, Toulouse, France; c CHU Rennes, Rennes University, LTSI UMR Inserm 1099, CIC Inserm 1414, 35000 Rennes, France; d Service de Cardiologie, Hôpital Louis Pradel, CHU de Lyon, Lyon, France; e Service de Chirurgie Cardiaque, Hôpital Louis Pradel, CHU de Lyon, Lyon, France; f Université Paris Cité, Service de cardiologie, Hôpital Bichat, APHP, Paris, France; g Service de Cardiologie, CHU de Dijon, Dijon, France; h Service de Cardiologie, CHU de Lille, Lille, France; i Service de Cardiologie, CHU de Grenoble, Grenoble, France; j Service de Cardiologie, CHU La Timone, Marseille, France; k Service de Cardiologie, Hôpital Européen Georges Pompidou, AP-HP, Paris, France; l Service de Cardiologie, CHU de Caen, Caen, France; m Service de Cardiologie, CHU de Nancy, Nancy, France; n Service de Chirurgie Cardiaque, CHU de Strasbourg, Strasbourg, France; ° Service de Cardiologie, CHU de Rouen, Rouen, France; p Electrophysiology Department, Hôpital Cardiologique du Haut Lévêque, Pessac, France; q Heart Failure Unit, Cardiology Department, Hôpital Cardiologique du Haut Lévêque, Pessac, France; r Assistance Publique-Hôpitaux de Paris, Pitié-Salpêtrière Hospital, Institute of Cardiology, Institute for Cardiometabolism and Nutrition, Paris, France

**Keywords:** Electrical storm, Heart transplantation, Heart failure, Cardiogenic shock, Ventricular assist device

## Abstract

**Background:**

Heart transplantation (HTx) has been suggested for refractory electrical storm (ES). Data regarding the outcomes of these patients are scarce.

**Objective:**

To compare the prognosis of refractory ES patients listed for urgent HTx, with and without transplantation.

**Methods:**

Patients registered on urgent HTx waiting list for refractory ES were retrospectively included in 13 French centers between 2010–2022. The primary endpoint was in-hospital all-cause mortality.

**Results:**

Eighty-five patients were included (85.9% men; 56.0 [48.0–61.0] years old; 55.1% with dilated cardiomyopathy), among whom 45 (52.9%) ultimately underwent HTx during index hospitalization. In the overall cohort, 89.3% of patients received amiodarone, 64.3% beta-blockers, 45.9% required deep sedation, 5.9% underwent stellate ganglion block, and 41.2% received mechanical circulatory support. Catheter ablation was less frequently performed in the transplanted group (20.0% vs 57.5%, p < 0.01). No difference was found for in-hospital mortality between transplanted and non-transplanted patients (28.9% vs 35.0%, HR 0.72 [0.34–1.53], p=0.0.39). After 1-year follow-up, 14 patients (16.5%) of the non-transplanted group eventually underwent HTx, with 4 of them dying subsequently. Five patients (5.9%) were removed from HTx waiting list due to functional improvement. Conversely, 14 transplanted patients (16.5%) died.

**Conclusion:**

Refractory ES carries a high in-hospital mortality rate, affecting one-third of patients. Overall, 69.4% of patients listed for urgent HTx underwent transplantation, including 52.9% during index hospitalization and 16.5% within the year post-discharge, highlighting the need to optimize selection criteria and implement a comprehensive treatment that sometimes allows overcoming the acute phase, enabling HTx under more stable conditions.

## Graphical abstract

**Figure:**
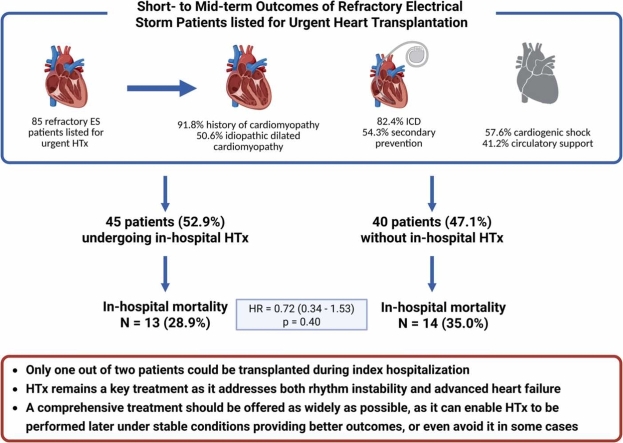

## Introduction

Electrical storm (ES) is defined as three or more episodes of sustained ventricular arrhythmias (VAs) occurring within 24 h, separated by at least 5 min, each requiring termination by an intervention like antiarrhythmic drugs, anti-tachycardia pacing or cardioversion/defibrillation.^1^, ^2^ Accordingly, the clinical presentation can range from asymptomatic/mildly symptomatic episodes of well-tolerated ventricular tachycardia (VT) to a life-threatening electrical instability.^3^ Most patients experiencing refractory ES generally suffer from pre-existing heart failure (HF) evolving concurrently, in which the onset of an ES notably worsens the prognosis, indicative of an ongoing progressive decline in myocardial function, negatively affecting patients’ quality of life and outcomes.^4^

In case of intractable electrical instability despite optimal treatment, urgent heart transplantation (HTx) has emerged as a standalone therapeutic alternative.^2^, ^5^ Although serving as a bail-out therapy, its wide implementation is hindered by 1/ the scarcity of donors, contrasting with an increasing number of potential recipients^6^ 2/ a substantial post-operative mortality related to complications such as acute rejection or extracardiac issues (sepsis, stroke)^5^, ^7^ and 3/ inferior survival outcomes when performed urgently compared to elective procedures.^8^ Consequently, a significant proportion of refractory ES patients may not benefit from urgent HTx, either due to the unavailability of a suitable donor or death before donor availability.^9^, ^10^ For all these reasons, the cornerstone of refractory ES management would be to optimally select patients for whom the expected outcomes of urgent HTx will be maximized, by carefully weighing on one hand, the HTx-related mortality risk factors and, on the other hand, the accessibility and comprehensiveness of electrophysiological treatment.

Given the scarcity of data on this topic, we conducted this multicentre study to compare the prognosis of patients managed for refractory ES listed for HTx who finally did and did not undergo urgent HTx.

## Materials and methods

### Patient population

This observational, retrospective, multicentre study aims to investigate the characteristics and outcomes of patients listed for urgent HTx after refractory ES across 13 French tertiary centers from 2010 to 2022. Patients were included if they experienced an ES and were subsequently listed for HTx or if they were already registered before ES due to their underlying cardiomyopathy, but switched to or prioritized to urgent list due to the occurrence of refractory ES. Throughout the entire study period (2010–2022), the French organ allocation system allowed patients with refractory ES to be prioritized for urgent HTx, without major changes in allocation rules regarding this indication.

### Data collection and follow-up

Baseline data encompassed demographic characteristics, cardiovascular risk factors (smoking, hypertension, dyslipidaemia, diabetes mellitus), history of HF (cardiomyopathy type and duration), left ventricular ejection fraction (LVEF), prior VAs, and implantable cardioverter-defibrillator (ICD) implantation status. In-hospital information included ES treatments (antiarrhythmic drugs, catheter ablation, invasive ventilation, acute mechanical circulatory support [aMCS], stellate ganglion blockade), clinical and laboratory parameters, and ES triggers, sourced from medical records. Long-term follow-up adhered to local hospital protocols.

### Endpoints

The primary endpoint was in-hospital all-cause mortality, while the secondary endpoint was 1-year all-cause mortality. Causes of death were additionally explored when available.

### Statistical analysis

Continuous variables are reported as medians and interquartile ranges (IQR). Categorical variables are described as frequencies and percentages. Comparisons were made using Mann Whitney non-parametric test for continuous variables and chi-square test or Fisher's exact test for categorical variables. Primary outcome of all-cause mortality was assessed using Kaplan-Meier time-to-event analysis, and time-varying Cox proportional hazards models were used to account for immortal time bias. Multivariable Cox models adjusted for age, sex, history of VAs, known cardiomyopathy, ICD, use of aMCS, and catheter ablation were used to determine the hazard ratio (HR) with 95% confidence interval (CI) and p values. In addition to the in-hospital analysis, a 1-year survival analysis was performed. In this analysis, patients in the non-transplanted group were censored at the time of HTx or VAD implantation if these events occurred after hospital discharge. Furthermore, a landmark analysis was conducted at day 30, restricted to patients who were alive at that time, to assess post-acute survival and minimize early treatment-related bias. Three exploratory analyses were conducted: (1) comparing patients in cardiogenic shock at admission, (2) comparing patients listed for HTx before ES onset versus others, (3) comparing those who underwent catheter ablation versus those who did not.

All tests were two-tailed. A value of p ≤ 0.05 was accepted as statistically significant. Analyses were performed using R software (version 4.3.2 (2023–10-3)).

### Ethics

All patients received information about anonymized data collection according to French ethics and regulatory law (Public Health Code). This study was approved by the local ethic committee. Written informed consent for participation was not required for this study in accordance with the national legislation. The study was registered by the Toulouse University Hospital (registration number: RnIPH 2024–71-59) and covered by the MR-004. All methods were carried out in accordance with relevant French national guidelines and regulations.

## Results

### Baseline characteristics

Eighty-five patients were enrolled in the study, with their characteristics summarized in Table 1. The median age of the cohort was 56.0 years (48.0 - 61.0), comprising 73 male patients (85.8%). Only 7 patients (8.2%) had no history of cardiomyopathy prior to ES. Idiopathic dilated cardiomyopathy (IDCM) was the most frequent underlying heart disease, observed in 43 patients (55.1%), while ischemic cardiomyopathy (ICM) was noted in 20 patients (25.6%). Of note, within the IDCM subgroup, pathogenic mutations in LMNA (7 patients), DSP (2 patients), and MYH7 (1 patient) were identified. Moreover, all five patients with hypertrophic cardiomyopathy and five of the six patients with arrhythmogenic cardiomyopathy were in the transplanted group. The median latest known LVEF was 27.0% (20.0 – 35.0). Of the cohort, 70 patients (82.4%) had an ICD, with 38 implanted for secondary prevention (54.3%), and 53 patients (62.4%) had a history of VT. Before ES onset, 65 patients (77.4%) were receiving beta-blocker therapy, while 38 patients (45.2%) were prescribed amiodarone. An initial presentation of cardiogenic shock was reported in 49 patients (57.6%), with an admission median LVEF of 20.0% (15.0 – 30.0), as shown in Table 2.

**Table 1:** Baseline Characteristics of the Study Population

	Overall Population (n = 85)	Patients Transplanted (n = 45)	Patients Not Transplanted (n = 40)	p Value
Age, years, median (IQR)	56.0 (48.0 – 61.0)	55.0 (47.8 – 59.3)	56.5 (50.0 – 61.3)	0.34
Male sex, n (%)	73 (85.9)	37 (82.2)	36 (90.0)	0.36
BMI, kg/m², median (IQR)	26.4 (23.5 – 29.2) (n = 83)	26.2 (23.4 – 28.6)	26.7 (24.2 – 29.3) (n = 38)	0.64
Body surface area, m², median (IQR)	1.99 (1.86 – 2.12) (n = 83)	1.97 (1.85 – 2.07)	2.00 (1.86 – 2.18) (n = 38)	0.25
Cardiovascular risk factors, n (%)				
Current smoking	26 (30.6)	17 (37.8)	9 (22.5)	0.16
Dyslipidaemia	28 (32.9)	10 (22.2)	18 (45.0)	0.04
Hypertension	30 (35.3)	17 (37.8)	13 (32.5)	0.65
Diabetes mellitus	15 (17.6)	6 (13.3)	9 (22.5)	0.39
History of cardiomyopathy, n (%)	78 (91.8)	45 (100.0)	33 (82.5)	0.12
Ischemic	20 (25.6)	12 (26.7)	8 (24.2)	
Non-ischemic dilated cardiomyopathy	43 (55.1)	22 (48.9)	21 (63.6)	
Cardiac sarcoidosis	1 (1.3)	0 (0.0)	1 (3.0)	
Arrhythmogenic	6 (7.7)	5 (11.1)	1 (3.0)	
Hypertrophic	5 (6.4)	5 (11.1)	0 (0.0)	
Congenital	1 (1.3)	1 (2.2)	0 (0.0)	
Unknown	2 (2.6)	0 (0.0)	2 (6.1)	
Time from cardiomyopathy diagnosis to ES, n (%)				0.76
< 6 months	5 (6.4)	2 (4.4)	3 (9.1)	
6 months – 5 years	11 (14.1)	6 (13.3)	5 (15.2)	
> 5 years	62 (79.5)	37 (82.2)	25 (75.8)	
Latest known LVEF, %, median (IQR)	27.0 (20.0 – 35.0)	25.0 (20.0 – 34.3)	29.0 (20.0 – 35.0)	0.89
Usual treatment prior to ES, n (%)				
Betablockers	65 (77.4) (n = 84)	40 (88.9)	25 (64.1) (n = 39)	< 0.01
Amiodarone	38 (45.2) (n = 84)	22 (48.9)	16 (41.0) (n = 39)	0.52
ACEI/ARB	41 (49.4) (n = 83)	25 (55.6)	16 (42.1) (n = 38)	0.27
Sacubitril/valsartan	21 (25.3) (n = 83)	11 (24.4)	10 (26.3) (n = 38)	1.00
MRA	40 (48.2) (n = 83)	19 (42.2)	21 (55.3) (n = 38)	0.27
Loop diuretics	53 (63.9) (n = 83)	31 (68.9)	22 (57.9) (n = 38)	0.36
Anticoagulant	41 (48.8) (n = 84)	25 (55.6)	16 (41.0) (n = 39)	0.20
Antiplatelet agents	24 (28.9) (n = 83)	11 (24.4)	13 (34.2) (n = 38)	0.34
Statin	29 (34.9) (n = 83)	13 (28.9)	16 (42.1) (n = 38)	0.25
Ventricular arrythmias, n (%)				
Ventricular tachycardia	53 (62.4)	28 (62.2)	25 (62.5)	1.00
Ventricular fibrillation	10 (11.8)	5 (11.1)	5 (12.5)	1.00
Electrical storm	27 (31.8)	12 (26.7)	15 (37.5)	0.35
VT ablation	28 (32.9)	12 (26.7)	16 (40.0)	0.25
ICD, n (%)	70 (82.4)	41 (91.1)	29 (72.5)	0.04
Secondary prevention	38 (54.3)	21 (51.2)	17 (58.6)	
Primary prevention	32 (45.7)	20 (48.8)	12 (41.4)	
Single chamber	16 (22.9)	10 (24.4)	6 (20.7)	
Dual chamber	19 (27.1)	8 (19.5)	11 (37.9)	
Resynchronization therapy	25 (35.7)	13 (31.7)	12 (41.4)	

ACEI/ARB, angiotensin-converting enzyme inhibitor/angiotensin receptor blocker; BMI, body mass index; IQR, interquartile range; MRA, mineralocorticoid receptor antagonist

**Table 2:** Clinical, Echocardiographic, and Biological Findings at Baseline

	Overall Population (n = 85)	Patients Transplanted (n = 45)	Patients Not Transplanted (n = 40)	p Value
Cardiogenic shock, n (%)	49 (57.6)	28 (62.2)	21 (52.5)	0.39
Blood tests at admission, median (IQR)				
Sodium, mmol/L	137.0 (134.0 – 140.0) (n = 73)	137.0 (134.0 – 139.8) (n = 34)	137.0 (135.0 – 139.5) (n = 39)	0.59
Creatinine, µmol/L	105.0 (87.8 – 136.0) (n = 76)	106.0 (93.5 – 150.0) (n = 36)	103.5 (80.8 – 132.5)	0.31
Bilirubin, mg/L	13.5 (8.2 – 20.0) (n = 62)	14.0 (8.8 – 24.8) (n = 29)	13.0 (8.0 – 19.6) (n = 33)	0.45
Arterial blood lactates, mmol/L	1.3 (1.0 – 1.8) (n = 48)	1.2 (1.0 – 2.2) (n = 17)	1.4 (1.0 – 1.8) (n = 31)	0.90
LVEF, %, median (IQR)	20.0 (15.0 – 30.0)	20.0 (15.0 – 25.0)	21.0 (20.0 – 30.0)	0.09

IQR, interquartile range, LVEF, left ventricular ejection fraction.

### Characteristics and management of the causal ES

Table 3 provides an overview of the characteristics and management strategies employed for ES. Nineteen patients (22.4%) encountered a triggering event, with ST-segment elevation myocardial infarction (STEMI) being the primary occurrence. After ES onset, the predominant antiarrhythmic medications administered included amiodarone (89.3%), beta-blockers (64.3%), magnesium sulfate (50.0%), and lidocaine (44.0%). Despite medications, ES management required deep sedation in 39 patients (45.9%), stellate ganglion blockade in 5 patients (5.9%), and aMCS in 35 patients (41.2%), primarily through ECMO (n = 28, 80.0%). VT ablation was performed in 32 patients (37.6%).

**Table 3:** Electrical Storm Characteristics and Management in the Overall Population

	Overall Population (n = 85)	Patients Transplanted (n = 45)	Patients Not Transplanted (n = 40)	p Value
Initial reason for hospitalization, n (%)				0.01
Ventricular arrythmia (VT, VF, ES, syncope, ICD shock)	67 (78.8)	41 (91.1)	26 (65.0)	
STEMI	5 (5.9)	1 (2.2)	4 (10.0)	
NSTEMI	0 (0.0)	0 (0.0)	0 (0.0)	
Cardiogenic shock, heart failure	13 (15.3)	3 (6.7)	10 (25.0)	
Trigger factor, n (%)				0.35
STEMI	7 (8.2)	1 (2.2)	6 (15.0)	
NSTEMI	3 (3.5)	2 (4.4)	1 (2.5)	
Hypokalaemia	4 (4.7)	3 (6.7)	1 (2.5)	
Infection	2 (2.4)	1 (2.2)	1 (2.5)	
Hyperthyroidism	2 (2.4)	1 (2.2)	1 (2.5)	
VT ablation	1 (1.2)	1 (2.2)	0 (0.0)	
None	66 (77.6)	36 (80.0)	30 (75.0)	
Anti-arrhythmic drugs, n (%)				
Betablocker	54 (64.3) (n = 84)	29 (64.4)	25 (64.1) (n = 39)	1.00
Amiodarone	75 (89.3) (n = 84)	42 (93.3)	33 (84.6) (n = 39)	0.29
Lidocaine	37 (44.0) (n = 84)	21 (46.7)	16 (41.0) (n = 39)	0.66
Magnesium sulfate	42 (50.0) (n = 84)	22 (48.9)	20 (51.3) (n = 39)	1.00
Deep sedation, n (%)	39 (45.9)	19 (42.2)	20 (50.0)	0.52
Stellate ganglion blockade, n (%)	5 (5.9)	1 (2.2)	4 (10.0)	0.18
VT ablation, n (%)	32 (37.6)	9 (20.0)	23 (57.5)	< 0.01
Time from ES to ablation, days, median (IQR)	4.0 (2.0 – 8.5)	3.0 (2.0 – 5.0)	5.0 (2.5 – 12.5)	< 0.01
Endo-epicardial procedure, n (%)	9 (34.6) (n = 26)	2 (22.2)	7 (41.2) (n = 17)	0.42
Noninducibility of VT after ablation, n (%)	10 (38.5) (n = 26)	4 (44.4)	6 (35.3) (n = 17)	0.69
≥ 2 different VT morphologies induced	17 (65.4) (n = 26)	6 (66.7)	11 (64.7) (n = 17)	0.25
Vasoactive and inotrope agents, n (%)				
Dobutamine	39 (45.9)	19 (42.2)	20 (50.0)	0.66
Norepinephrine	18 (21.2)	10 (22.2)	8 (20.0)	0.79
Epinephrine	5 (5.9)	1 (2.2)	4 (10.0)	0.19
Acute mechanical circulatory support, n (%)	35 (41.2)	22 (48.9)	13 (32.5)	0.19
ECMO	28 (80.0)	16 (72.7)	12 (92.3)	
IABP	7 (20.0)	6 (27.3)	1 (7.7)	

ECMO, extracorporeal membrane oxygenation; ES, electrical storm; IABP, intra-aortic balloon pump; NSTEMI, Non-ST-segment elevation myocardial infarction; SD, standard deviation; STEMI, ST-segment elevation myocardial infarction; VF, ventricular fibrillation; VT, ventricular tachycardia

### Comparison between ES patients receiving and not receiving urgent HTx during index hospitalization

Among the 85 patients included for refractory ES listed for urgent HTx, 45 (52.9%) ultimately underwent transplantation during index hospitalization. The median time from the onset of ES to transplant waitlist registration was significantly shorter in the transplanted group (3.0 days vs. 14.0 days, p < 0.01). The median time from HTx listing to transplantation was 5.0 days (2.0–13.3 days). Transplanted ES patients exhibited comparable baseline characteristics to non-transplanted ones, except for a higher prevalence of prior beta-blocker therapy (88.9% vs 64.1%, p < 0.01) and previous ICD implantation (91.1% vs 72.5%, p = 0.04). Besides, the distribution of cardiomyopathy subtypes was similar between the two groups, with a common predominance of IDCM (Table 1). There were no significant differences observed regarding initial clinical or laboratory parameters, nor for baseline LVEF (20.0% vs 21.0%, p = 0.09) (Table 2).

However, transplanted patients notably less frequently underwent VT ablation (20.0% vs 57.5%, p < 0.01) during index hospitalization. Otherwise, there were no substantial differences in the use of amiodarone (93.3% vs 84.6%, p = 0.29), beta-blockers (64.4% vs 64.1%, p = 1.00), or deep sedation (42.2% vs 50.0%, p = 0.52) or aMCS (48.9% vs 32.5%, p = 0.19). The use of vasopressors/inotropes was also similar between the groups, with numerically less use of stellate ganglion blockade in transplanted patients (2.2% vs 10.0%, p = 0.18) (Table 3).

### Short-term outcomes

Thirteen (28.9%) of the 45 transplanted patients died in-hospital, primarily from non-cardiac causes (n = 10, 76.9%). The two leading causes of in-hospital deaths were pulmonary sepsis (n = 5, 38.5%) and cerebral hemorrhages (n = 3, 23.1%). During the same period, the all-cause in-hospital mortality rate among all transplanted patients in the included centers was 13.2%.

In comparison, 14 of the 40 non-transplanted patients (35.0%) died in-hospital. Four of them had undergone ventricular assistance device (VAD) implantation and died subsequently due to non-cardiac causes (three sepsis and one cerebral hemorrhage). Among the remaining 10 patients, five died from non-cardiac causes (three sepsis, one hemorrhagic digestive shock, and one of unknown cause), two from electrical instability, and two from end-stage HF (one cause unknown).

Kaplan–Meier survival analysis revealed no significant difference in all-cause in-hospital mortality between transplanted and non-transplanted patients (HR 0.72 [0.34 - 1.53], p = 0.39). Interestingly, when restricting the analysis to patients who initially presented with cardiogenic shock, in-hospital mortality is significantly lower for those who underwent HTx (25.0% vs. 66.7%, HR 0.24 [0.10 - 0.61], p < 0.01). Additionally, non-transplanted patients were more frequently hospitalized at baseline for cardiogenic shock/acute HF (6.7% vs. 25.0%, p < 0.01), and those in cardiogenic shock showed a trend toward a lower rate of catheter ablation (28.6% vs. 50.0%, p = 0.07).

### Mid-term outcomes

**Figure 1:**
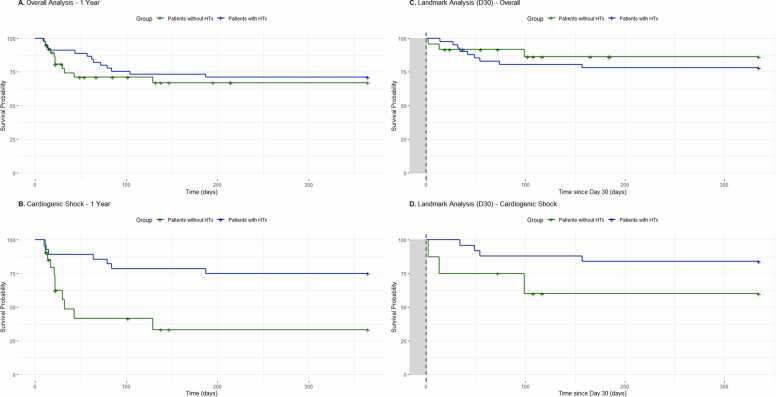
One-year survival and landmark analysis in refractory electrical storm patients listed for urgent heart transplantation. Kaplan-Meier survival curves comparing transplanted versus non-transplanted patients. A. One-year survival in the overall cohort. B. One-year survival in patients with cardiogenic shock. C. Landmark analysis at day 30 in the overall cohort, restricted to patients alive at that timepoint (gray shaded area represents the landmark period from day 0 to day 30). D. Landmark analysis at day 30 in patients with cardiogenic shock. HTx, heart transplantation.

At one-year follow-up, survival analysis revealed no significant difference in mortality between transplanted and non-transplanted patients in the overall population (HR 1.24 [0.41–3.76], p = 0.70). However, among patients presenting with cardiogenic shock, those who underwent HTx demonstrated significantly lower mortality compared to non-transplanted patients (HR 0.19 [0.05–0.71], p = 0.01) (Figure 1). A landmark analysis conducted at day 30, restricted to patients alive at that time, yielded similar results. In the overall population, no significant mortality difference was observed between groups (HR 1.06 [0.23–3.55], p = 0.94), whereas patients with cardiogenic shock who underwent HTx continued to show improved survival (HR 0.71 [0.45–0.98], p = 0.04).

**Figure 2:**
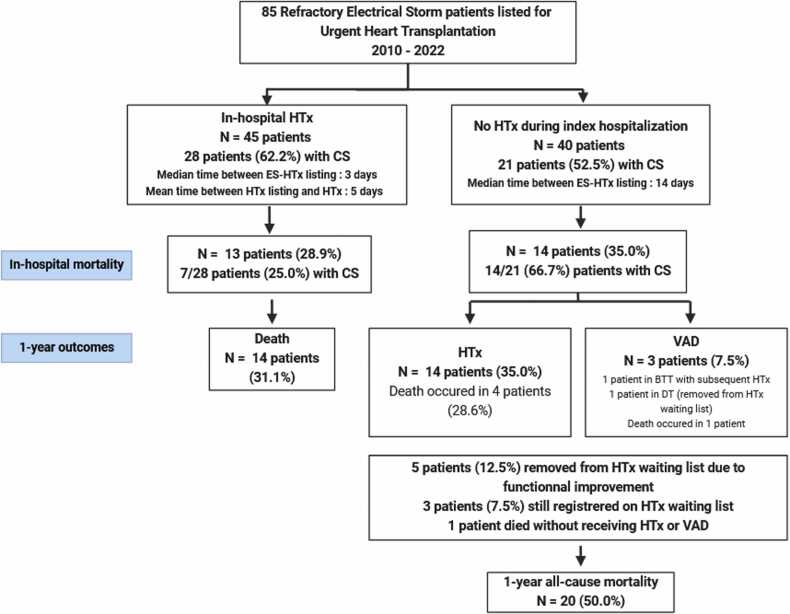
Short and Mid Term Outcomes of Refractory Electrical Storm Patients Listed for Urgent Heart Transplantation. BTT, bridge-to-transplant; CS, cardiogenic shock; DT, destination therapy; ES, electrical storm; HTx, heart transplantation; VAD, ventricular assist device.

Within the non-transplanted group, 14 patients (35.0% overall, 53.8% of patients discharged from index hospitalization) eventually underwent HTx after 1 year of follow-up (including 11 more than one month after the index hospitalization), with 4 of them dying subsequently. Additionally, 3 patients (7.5%) underwent VAD implantation—one as a bridge-to-transplant strategy who was subsequently transplanted, one as a destination therapy strategy, and the third resulting in immediate post-operative death. All these three patients had an ICD and a history of ischemic cardiomyopathy, with none already listed for HTx. Two of them presented initially with cardiogenic shock and STEMI-triggered ES, while the last was triggered by an infection. An endocardial ablation was performed on two of them, with only one showing non-inducibility of VT after the procedure. One patient died without previous HTx or VAD (unknown etiology). Eventually, 5 patients (12.5%) were removed from the HTx waiting list due to significant functional improvement, while 3 patients (7.5%) remained listed and awaiting a donor graft. Conversely, death occurred in 14 patients of the transplanted group (31.1%), with only one of these deaths occurring after discharge from the hospital in this group (Figure 2).

### Comparison between patients previously registered on the HTx waiting list and others

Overall, 10 patients (11.8%) were already registered on the HTx waiting list before ES onset due to their underlying cardiomyopathy, with no significant differences in nearly all baseline characteristics or ES treatment modalities (Supplemental Tables 1, 2, and 3), albeit with a small sample size. Among these 10 patients, only one (10.0%) underwent an ablation procedure, while 9 (90.0%) ultimately underwent transplantation during index hospitalization. Four of these patients (40.0%) died in-hospital (3 in the transplanted group and the non-transplanted patient), with no significant difference compared to the non-listed group (40.0% vs. 30.7%, HR 1.39 [0.48 - 4.03], p = 0.54), albeit with a wide confidence interval. The six remaining transplanted patients who were discharged were all alive at one-year follow-up.

### Features and outcomes of ablation procedure

Overall, the median time between ES onset and ablation was 4.0 (2.0 – 8.5) days (Table 1), which was significantly shorter in the transplanted group (3.0 vs. 5.0 days, p < 0.01). All procedures were performed using an endocardial approach, except for 9 patients (34.6%) who underwent a combined endo-epicardial procedure, with no significant difference between transplanted and non-transplanted groups. More than two different VT morphologies were induced in 17 patients (65.4%), and 10 patients (38.5%) were non-inducible at the end of the procedure, with similar rates between groups. Ultimately, patients treated with ablation presented with less severe baseline characteristics, as reflected by a slightly higher LVEF (22.5% vs. 20.0%, p = 0.05), a lower rate of admission for HF/cardiogenic shock (3.1% vs. 22.6%, p = 0.02), and a lower median bilirubin level (11.7 vs. 16.0 mg/L, p < 0.01) (Supplemental Tables 4, 5, and 6).

## Discussion

To date, this is the first study to assess the prognosis of such a large number of patients listed for urgent HTx due to refractory ES and to evaluate the impact of HTx on mortality outcomes. Our main findings indicate that: 1/ one out of two patients listed for urgent HTx couldn’t be transplanted due to donor scarcity; 2/ patients in the transplanted group were treated less aggressively from an electrophysiological standpoint, notably illustrated by a lower rate of VT catheter ablation; 3/ we did not observe a difference in in-hospital mortality between patients who underwent urgent HTx and those who did not; 4/ one out of two patients who were not transplanted during the index hospitalization will eventually undergo transplantation within the year following the initial electrical storm and 5/ 12.5% of patients who did not undergo HTx in the acute phase were subsequently withdrawn the list due to sufficient functional improvement.

Although occasionally performed in the context of refractory ES,^5^ the exact role of urgent HTx remains relatively unclear and is not addressed in the latest 2022 ESC guidelines on the management of VA^1^ or in the latest guidelines focusing on ES management.^2^ One of the main crucial issues lies in the lack of precision regarding optimal patient selection criteria for whom HTx would represent the best possible option in both the short and long terms, from both an electrical and HF standpoint.

Indeed, the unfavorable prognostic impact resulting from the occurrence of an ES in the progression of HF is extensively documented, leading to a decline in quality of life and an increased combined risk of death and hospitalization for decompensated HF.^11^ Most ES episodes occur in the setting of advanced heart disease,^2^ with a pre-existing significant impairment of LVEF, as observed in our study. Therefore, the occurrence of a refractory ES urges to simultaneous evaluation and treatment not only of acute electrical instability but also of the subsequent functional prognosis of HF. This logically prompts consideration of urgent HTx as a potential rescue solution for both issues.

In our study, out of the 85 patients urgently listed for HTx due to refractory ES, approximately half were finally transplanted during index hospitalization (45 patients, 52.9%). Despite being listed in the same way, the remaining 40 patients could not undergo urgent HTx due to either a lack of suitable donor offers or death before being transplanted. This low rate may seem somewhat disappointing, as the French organ allocation system grants the maximum number of points to patients listed with ECMO-type aMCS and/or refractory ES.^12^ Several factors may explain this outcome, foremost among them 1) the scarcity of donors^6^ and 2) the multiple organ failures frequently associated in this critical situation, which can lead to death before an appropriate graft is offered, or temporary removal from the list for severe multiorgan failure if the estimated risk of post-transplantation death within 1 year exceeds 50%.^13^ Nevertheless, a significant proportion of patients who could not be transplanted were eventually discharged from the hospital. All of these data support the broadest possible implementation of the full range of interventional treatment for refractory ES, given that only half of the patients listed for transplantation were ultimately transplanted. Furthermore, this highlights the crucial need to optimize selection criteria for urgent HTx in the context of refractory ES, in order to reserve it for the most extreme cases where the chances of myocardial and rhythm recovery are minimal. Similarly, LVADs, which can generally be used as bridge-to-transplant or destination therapy for patients suffering from isolated left ventricular dysfunction, do not appear suitable to these patients suffering from rhythm disorders, primarily due to increased mortality linked to the risk of right ventricular dysfunction in case of subsequent ventricular arrhythmia recurrence.^14^, ^15^ This is further supported in our cohort by the fact that, of the six LVADs implanted in-hospital, four patients died during the post-operative period. Yet, VAD could represent also a suitable option for selected patients, especially in cases of chronic cardiomyopathy complicated by ES onset triggered by acute reversible factors such as STEMI or infection, particularly when access to HTx is challenging, whether due to specific contraindications or the absence of a suitable donor offer in the context of organ shortage. Moreover, recent observational research^16^, ^17^ indicates that while recurrent ventricular arrhythmia is associated with worse survival in VAD recipients, VT ablation in this population has been correlated with improved outcomes compared to medical management alone, aligning survival rates with those of other VAD recipients without post-implant VT. Hence, further studies are needed to establish standardized guidelines for this specific population.

Interestingly, both groups exhibited globally similar baseline characteristics, although some significant differences were observed, including a higher prevalence of ICD use and beta blocker treatment prior to the onset of ES, as well as a trend towards higher rates of hypertrophic and arrhythmogenic cardiomyopathy in transplanted patients. Meanwhile, no significant differences were observed in the severity of initial admission, with similar proportions of cardiogenic shock and comparable median LVEF. However, a crucial point lies in the notably lower utilization of VT ablation in the transplanted patient group. Indeed, only 9 of them (20.0%) underwent VT ablation, which may seem surprisingly low, particularly in a population predominantly comprising patients with structural heart diseases managed in tertiary centers. Several factors may explain this relatively low rate: 1) a history of ablation in 26.7% of patients, for whom a redo procedure may have been canceled due to low expected benefit given the documented substrate anatomy during previous ablations; 2) Initial hemodynamic instability, which complicates the performance of a comprehensive ablation, increases the risk of further deterioration, and poses challenges for ventricular arrhythmia mapping, thereby discouraging the procedure; 3) The data collection period extending up to 2010, during which the availability and technical expertise in ablation procedures were less developed; 4) contraindications to catheter ablation, such as left ventricular thrombus. Although limited by small sample size, our data seem to support this hypothesis, as ablation appears to have been preferentially performed in relatively less severe patients (e.g., higher LVEF, lower rate of HF/cardiogenic shock, lower bilirubin level). Furthermore, we also hypothesize that this low rate of VT ablation is a consequence of the transplantation strategy adopted by some Heart Teams, prioritizing urgent HTx for these patients and therefore preferring to postpone VT ablation, possibly due to fears of potentially fatal complications. Indeed, VT ablation is an invasive procedure associated with inherent complications such as cardiac tamponade, stroke, or hemodynamic instability, with rates reaching up to 10% in ES patients,^18^, ^19^ which can further worsen the prognosis. This hypothesis is partly reinforced by the operational rules of the French organ allocation system,^12^ wherein a situation of refractory ES and/or the need for ECMO assistance allow for the maximum allocation of points, thereby placing the patient at the top of the waiting list and prioritizing them. Our observational data appear to support this hypothesis, as among the 10 patients already listed for HTx before ES onset—representing the most severe cases of advanced HF—only one underwent an ablation procedure (and was still transplanted in-hospital), whereas nine underwent transplantation during the index hospitalization. Similarly, patients with a history of cardiomyopathy were also less frequently treated with ablation. The exact role of ablation in this context remains to be clarified. Rather than serving as an alternative to HTx, ablation could sometimes facilitate transplantation under better hemodynamic conditions by stabilizing electrical instability in cases of manifest HF deterioration. Alternatively, in cases where the instability was purely electrophysiological, ablation might help delay HTx or provide a more accurate reassessment of HF severity, better reflecting the patient’s true underlying condition. Conversely, VT ablation use was significantly higher in the non-transplanted group, albeit with room for improvement, in which 23 patients (57.5%) underwent the procedure, with redo procedures performed for 11 of them, indicating a trend to optimize interventional treatment in the absence of a graft offer. Moreover, the median time between ES onset and ablation was longer in non-transplanted patients, suggesting an initial 'watchful waiting' approach, where ablation was initially deferred, perhaps partly due to concerns about associated complications and while awaiting the patient's hemodynamic, arrhythmic, and HF progression. Beyond the chronic progression of the underlying cardiomyopathy, its acute presentation also introduces additional challenges, particularly in cases of cardiogenic shock. This situation is highly complex, as inodilators/vasopressors used for cardiogenic shock have pro-arrhythmic effects that may sustain ES, seemingly favoring an initial HTx-oriented approach. However, both catheter ablation^2^ and HTx^8^ are more difficult to perform in the setting of cardiogenic shock and potential associated organ failures, and HTx outcomes are significantly worse when performed under emergency conditions. In our study, non-transplanted patients were more frequently hospitalized for HF/cardiogenic shock, which may have further complicated management—either leading to patient death before transplantation or delaying catheter ablation or HTx due to concerns over poor outcomes in a state of severe hemodynamic instability. Within the French organ allocation system, all patients with refractory ES are assigned the highest priority status, placing them at the top of the national waiting list regardless of cardiogenic shock status.^20^ This allocation policy grants equal theoretical access to donor organs for all ES patients. Interestingly, our results suggest a paradox: although all ES patients receive equal priority, those with cardiogenic shock appear to derive the greatest survival benefit from HTx, whereas no significant benefit was observed in the overall ES population. This finding suggests that cardiogenic shock may identify a subgroup with both the highest baseline risk and the greatest potential benefit from urgent HTx. In the context of persistent donor organ scarcity, these observations could inform future optimization of patient selection criteria, potentially prioritizing ES patients with cardiogenic shock for urgent listing while pursuing comprehensive electrophysiological management in hemodynamically stable ES patients.

Overall, we did not observe a difference for in-hospital mortality between transplanted and non-transplanted refractory ES patients. Furthermore, the in-hospital mortality rate for patients transplanted during index hospitalization was 28.9%, which is slightly higher than the overall post-HTx in-hospital mortality during the same period (13.2%), once again highlighting the need to refine selection criteria for the most suitable candidates, albeit with room for improvement. Indeed, a recent study^21^ focusing on HTx for pediatric patients with malignant arrhythmias found a lower rate of short- and long-term mortality, with no difference between patients with and without arrhythmia. Despite significant recent advancements, HTx is still marked by high perioperative mortality linked to multiple complications such as acute rejection, sepsis, stroke, kidney failure, and right ventricular dysfunction,^5^, ^7^ when performed in emergency settings, particularly in the presence of aMCS.^8^ Importantly, in-hospital mortality among patients transplanted for ES was higher than overall post-HTx mortality in the participating centers. Most early deaths were non-cardiac, reflecting the extreme severity and systemic vulnerability of these patients at urgent listing. This likely reflects both the severity of refractory arrhythmic instability and advanced end-stage HFs in this population, often associated with multiorgan dysfunction and comorbidities, as well as the impact of prolonged critical care and the need for aMCS in a substantial proportion of patients. Several models for predicting the risk of postoperative complications have been developed, based on various parameters (age, diagnosis, type of mechanical support, ventilator support, estimated glomerular filtration rate, and total serum bilirubin),^22^ and should be utilized in assessing the risk-benefit balance before opting for HTx. Nevertheless, it is clear and unequivocal that despite the risk of complications, HTx remains a key option in cases of refractory hemodynamic and electrical instability despite the implementation of multiple available options (VT ablation, stellate ganglion block, deep sedation, temporary external pacing…). Hence, while understandable given the difficulties in anticipating and managing refractory ES, we can only regret such a low utilization rate of VT ablation in the transplanted group, as its benefits are now clearly established. For instance, results from the multicentre randomized PARTITA trial,^23^ designed to evaluate the benefit of ablation after the first ICD shock, confirmed that VT ablation was associated with a reduced risk of death or worsening HF. Similarly, the multicentre VANISH trial,^24^ comparing VT ablation versus escalation of antiarrhythmic drugs, found consistent results, illustrated by a decrease in the risk of death, ES, or appropriate ICD shock. However, no randomized trial to date specifically addresses the population included in our study, which presents additional hemodynamic constraints and myocardial decline compared to the patients enrolled in the PARTITA^23^ and VANISH^24^ trials. Hence, further large-scale prospective studies are warranted to optimize the management of these patients, particularly regarding the timing and modalities of catheter ablation*.* Although difficult to demonstrate based on this single study, in light of existing data, it can be assumed that a number of these patients could have possibly benefited from a comprehensive treatment approach, including VT ablation, stellate ganglion blockade, or cardiac sympathetic denervation, which can sometimes eliminate the ES substrate, offering the possibility to delay or avoid the urgent HTx strategy. Although they were successfully discharged from the hospital, half of the patients who were not transplanted during the index hospitalization were eventually transplanted within the first year, whether due to a recurrence of rhythm disorders or progression to end-stage HF. Thus, HTx remains the treatment of choice for patients with advanced HF and rhythm instability but performing it electively under more favorable conditions can lead to better outcomes. In a recent study,^25^ focusing on the outcomes of 34 patients managed for refractory ES requiring initial ECMO support, it was found that 18 of them (53.0%) were eventually weaned from ECMO and successfully discharged from the hospital, while only 10 (29.0%) underwent HTx (with 9 still alive at discharge). This tends to confirm that implementing maximalist medical treatment on both the electrical and hemodynamic fronts can extinguish the ES substrate, allowing for the consideration of further management under more favorable circumstances, and in some cases, even obviating the need for HTx (5 patients [5.9%] removed from HTx waiting list after 1-year follow-up due to functional improvement in our study). Another study^26^ focusing on the comparison between catheter ablation and advanced therapy for patients with severe HF and ES, which included 73 patients, revealed results quite similar to ours, with no difference in mortality between patients who underwent VT ablation and those who directly proceeded with advanced therapy, although heart transplant rates were high during subsequent follow-up.

On the other hand, as previously discussed, VT ablation carries a significant risk of complications, which may dissuade some from undergoing the procedure, particularly in cases of acute hemodynamic decompensation during catheter ablation, the probability of which can be estimated using the PAINESD Score.^27^ However, this risk might not be prohibitive and may warrant reconsideration of the prophylactic use of aMCS before ablation, which has shown promising outcomes in observational studies^28^ although randomized data are still lacking to support its widespread adoption in clinical practice. In a recent pilot case series of patients with advanced HF and VT-ES,^29^ a PAINESD score-guided multidisciplinary management approach, which included preprocedural hemodynamic optimization, evaluation for advanced HF therapy options, and prophylactic initiation of VA-ECMO, was associated with favorable short- and long-term mortality outcomes and effective VT control. Furthermore, the use of invasive hemodynamic monitoring—which has previously shown prognostic value both in the context of HTx^30^, ^31^ and in ES—might potentially help identify patients who may not tolerate extensive mapping and ablation, or who may require aMCS support but could also be at risk of being non-weanable from it post-procedurally. Further studies are warranted to explore this approach.

### Limitations and future directions

Major limitations include the retrospective nature of the data and a possible referral bias because of the recruitment from tertiary centers in France. Moreover, our cohort was predominantly composed of non-ischemic cardiomyopathies, with a minority having ICM. Nevertheless, this topic would benefit from further stratification based on subtypes of cardiomyopathy, as each present with specific characteristics.^32^ Indeed, the structural complexity of arrhythmogenic substrate characteristics in non-ischemic cardiomyopathies generally make ablation procedures more challenging in this setting^33^ due to extended and rapidly evolutive substrate, sometimes warranting a different approach, such as the use of other techniques like cardiac sympathetic denervation.^34^, ^35^ Conversely, their long-term post-HTx outcomes are relatively favorable,^36^ which may have led to a cautious approach, avoiding ablation due to its potential complications and limited expected benefits in order to preserve future transplant eligibility. However, definitive conclusions cannot be drawn given the small sample size and the absence of functional HF parameters such as peak oxygen consumption. This topic warrants further investigation in larger studies. Due to the retrospective design and the long inclusion period, we were unable to differentiate whether cardiogenic shock preceded or resulted from electrical storm in patients with coexisting shock at ES onset, which should be considered when interpreting these outcomes. Lastly, we were unable to specify the distribution of blood groups, which is an important variable in the graft allocation system.

## Conclusion

Refractory ES is associated with a high in-hospital mortality rate, affecting one-third of patients. Only 52.9% of patients listed for urgent HTx ultimately underwent transplantation, highlighting the need to optimize selection criteria. A comprehensive treatment (catheter ablation, deep sedation, circulatory support, etc.) should be offered as widely as possible to all patients as it sometimes provides the opportunity to overtake the acute phase, allowing HTx to be considered later under more stable conditions with better preoperative preparation.

## Sources of Funding

None.

## Data availability statement

The datasets used and/or analyzed during the current study are available from the corresponding author on reasonable request.

## Declaration of Competing Interest

The authors declare that they have no known competing financial interests or personal relationships that could have appeared to influence the work reported in this paper.

## Footnotes

Appendix A Supplemental data associated with this article can be found in the online version at doi:10.1016/j.jhlto.2026.100529.
