# Supplementary material for: Short- to mid-term outcomes of refractory electrical storm patients listed for urgent heart transplantation
Source: JHLT Open. 2026 Feb 28;12:100529. doi: 10.1016/j.jhlto.2026.100529 (PMC13010970; doi:10.1016/j.jhlto.2026.100529)
Supplement: Supplementary file 1 — Supplemental material [file mmc1.docx]

| **Supplementary Table 1. Baseline characteristics of the study population according to HTx listing status before CS onset.** | | | | |
| --- | --- | --- | --- | --- |
|  | Overall population (n = 85) | Patients already listed for HTx before ES (n = 10) | Patients listed for HTx at the time of ES (n = 75) | p value |
| Age, years, median (IQR)  Male sex, n (%)  BMI, kg/m², median (IQR)  Body surface area, m², median (IQR)  Cardiovascular risk factors, n (%)  Current smoking  Dyslipidaemia  Hypertension  Diabetes mellitus  History of cardiomyopathy, n (%)  Ischemic  Non-ischemic dilated cardiomyopathy  Cardiac sarcoidosis  Arrhythmogenic  Hypertrophic  Congenital  Unknown  Time from cardiomyopathy diagnosis to ES, n (%)  < 6 months  6 months – 5 years  > 5 years  Latest known LVEF, %, median (IQR)  Usual treatment prior to ES, n (%)  Betablockers  Amiodarone  ACEI/ARB  Sacubitril/valsartan  MRA  Loop diuretics  Anticoagulant  Antiplatelet agents  Statin  Ventricular arrythmias, n (%)  Ventricular tachycardia  Ventricular fibrillation  Electrical storm  VT ablation  ICD, n (%)  Secondary prevention  Primary prevention  Single chamber  Dual chamber  Resynchronization therapy | 56.0 (48.0 – 61.0)  73 (85.9)  26.4 (23.5 – 29.2) (n = 83)  1.99 (1.86 – 2.12) (n = 83)  26 (30.6)  28 (32.9)  30 (35.3)  15 (17.6)  78 (91.8)  20 (25.6)  43 (55.1)  1 (1.3)  6 (7.7)  5 (6.4)  1 (1.3)  2 (2.6)  5 (6.4)  11 (14.1)  62 (79.5)  27.0 (20.0 – 35.0)  65 (77.4) (n = 84)  38 (45.2) (n = 84)  41 (49.4) (n = 83)  21 (25.3) (n = 83)  40 (48.2) (n = 83)  53 (63.9) (n = 83)  41 (48.8) (n = 84)  24 (28.9) (n = 83)  29 (34.9) (n = 83)    53 (62.4)  10 (11.8)  27 (31.8)  28 (32.9)  70 (82.4)  38 (54.3)  32 (45.7)  16 (22.9)  19 (27.1)  25 (35.7) | 57.0 (55.3 – 59.0)  9 (90.0)  31.2 (26.7 – 33.5)  2.18 (2.04 – 2.26)  5 (50.0)  4 (40.0)  6 (60.0)  3 (30.0)  10 (100.0)  6 (60.0)  3 (30.0)  0 (0.0)  0 (0.0)  1 (10.0)  0 (0.0)  0 (0.0)  0 (0.0)  0 (0.0)  10 (100.0)  22.5 (18.5 – 29.5)  10 (100.0)  6 (60.0)  5 (50.0)  2 (20.0)  4 (40.0)  7 (70.0)  6 (60.0)  5 (50.0)  3 (30.0)  5 (50.0)  2 (20.0)  2 (20.0)  4 (40.0)  9 (90.0)  4 (44.4)  5 (55.6)  2 (22.2)  0 (0.0)  4 (44.4) | 55.0 (48.0 – 61.0)  64 (85.3)  26.1 (23.0- 28.6) (n = 73)  1.97 (1.85 – 2.09) (n = 73)  21 (28.0)  24 (32.0)  24 (32.0)  12 (16.0)  68 (90.7)  14 (20.6)  40 (58.8)  1 (1.5)  6 (8.8)  4 (5.9)  1 (1.5)  2 (2.9)  5 (7.4)  11 (16.2)  52 (76.5)  29.0 (22.0 – 35.5)  55 (74.3) (n = 74)  32 (43.2) (n = 74)  36 (49.3) (n = 73)  19 (26.0) (n = 73)  36 (49.3) (n = 73)  46 (63.0) (n = 73)  35 (47.3) (n = 74)  19 (26.0) (n = 73)  26 (35.6) (n = 73)  48 (64.0)  8 (10.7)  25 (33.3)  24 (32.0)  61 (81.3)  34 (55.7)  27 (44.3)  14 (23.0)  19 (31.1)  21 (34.4) | 0.73  1.00  0.03  0.01  0.27  0.72  0.16  0.37  0.14  0.42  0.09  0.11  0.34  1.00  1.00  0.74  0.74  0.52  0.14  1.00  0.49  0.33  0.49  0.72  0.68 |

ACEI/ARB, angiotensin-converting enzyme inhibitor/angiotensin receptor blocker; BMI, body mass index; IQR, interquartile range; MRA, mineralocorticoid receptor antagonist

| **Supplementary Table 2. Clinical, echocardiographic, and biological findings at baseline according to HTx listing status before CS onset.** | | | | |
| --- | --- | --- | --- | --- |
|  | Overall population (n = 85) | Patients already listed for HTx before ES (n = 10) | Patients listed for HTx at the time of ES (n = 75) | p value |
| Cardiogenic shock, n (%)  Blood tests at admission, median (IQR)  Sodium, mmol/L  Creatinine, µmol/L  Bilirubin, mg/L  Arterial blood lactates, mmol/L  LVEF, %, median (IQR) | 49 (57.6)  137.0 (134.0 – 140.0) (n = 73)  105.0 (87.8 – 136.0) (n = 76)  13.5 (8.2 – 20.0) (n = 62)  1.3 (1.0 – 1.8) (n = 48)  20.0 (15.0 – 30.0) | 6 (60.0)  136.0 (134.5 – 139.5) (n = 7)  152.0 (119.5 – 183.5) (n = 7)  24.0 (11.0 – 67.0) (n = 5)  2.0 (1.3 – 1.5) (n = 2)  20.0 (15.0 – 27.5) | 43 (57.3)  137.0 (134.0 – 139.8) (n = 66)  102.0 (87.0 – 129.0) (n = 69)  13.0 (8.0 – 20.0) (n = 57)  1.3 (0.9 – 1.9) (n = 46)  20.0 (16.0 – 30.0) | 1.00  0.82  0.06  0.21  0.93  0.43 |

IQR, interquartile range, LVEF, left ventricular ejection fraction.

| **Supplementary Table 3. Electrical storm characteristics and management in the overall population according to HTx listing status before CS onset.** | | | | |
| --- | --- | --- | --- | --- |
|  | Overall population (n = 85) | Patients already listed for HTx before ES (n = 10) | Patients listed for HTx at the time of ES (n = 75) | p value |
| Initial reason for hospitalization, n (%)  Ventricular arrythmia (VT, VF, ES, syncope, ICD shock)  STEMI  NSTEMI  Cardiogenic shock, heart failure  Trigger factor, n (%)  STEMI  NSTEMI  Hypokalaemia  Infection  Hyperthyroidism  VT ablation  None  Anti-arrhythmic drugs, n (%)  Betablocker  Amiodarone  Lidocaine  Magnesium sulfate  Deep sedation, n (%)  Stellate ganglion blockade, n (%)  VT ablation, n (%)  Time from ES to ablation, days, median (IQR)  Endo-epicardial procedure, n (%)  Noninducibility of VT after ablation, n (%)  ≥ 2 different VT morphologies induced  Vasoactive and inotrope agents, n (%)  Dobutamine  Norepinephrine  Epinephrine  Acute mechanical circulatory support, n (%)  ECMO  IABP | 67 (78.8)  5 (5.9)  0 (0.0)  13 (15.3)  7 (8.2)  3 (3.5)  4 (4.7)  2 (2.4)  2 (2.4)  1 (1.2)  66 (77.6)  54 (64.3) (n = 84)  75 (89.3) (n = 84)  37 (44.0) (n = 84)  42 (50.0) (n = 84)  39 (45.9)  5 (5.9)  32 (37.6)  4.0 (2.0 – 8.5)  9 (34.6) (n = 26)  10 (38.5) (n = 26)  17 (65.4) (n = 26)  39 (45.9)  18 (21.2)  5 (5.9)  35 (41.2)  28 (80.0)  7 (20.0) | 9 (90.0)  0 (0.0)  0 (0.0)  1 (10.0)  0 (0.0)  0 (0.0)  1 (10.0)  1 (10.0)  0 (0.0)  8 (80.0)  0 (0.0)  7 (70.0)  8 (80.0)  3 (30.0)  2 (20.0)  2 (20.0)  0 (0.0)  1 (10.0)  3  0 (0.0)  1 (100.0)  0 (0.0)  5 (50.0)  1 (10.0)  0 (0.0)  2 (20.0)  2 (100.0)  0 (0.0) | 58 (77.3)  5 (6.7)  0 (0.0)  12 (16.0)  7 (9.3)  3 (4.0)  3 (4.0)  1 (1.3)  2 (2.7)  58 (77.3)  1 (1.3)  47 (63.5) (n = 74)  67 (90.5) (n = 74)  34 (45.9) (n = 74)  40 (54.1) (n = 74)  37 (49.3)  5 (6.7)  31 (41.3)  4.0 (2.0 – 8.5)  9 (36.0) (n = 25)  9 (36.0) (n = 25)  16 (64.0) (n = 25)  34 (45.3)  17 (22.7)  5 (6.7)  33 (44.0)  26 (78.8)  7 (21.2) | 1.00  0.46  1.00  0.29  0.50  0.09  0.11  1.00  0.08  NA  NA  NA  NA  1.00  0.68  1.00  0.19 |

ECMO, extracorporeal membrane oxygenation; ES, electrical storm; IABP, intra-aortic balloon pump; NSTEMI, Non-ST-segment elevation myocardial infarction; STEMI, ST-segment elevation myocardial infarction; VF = ventricular fibrillation; VT = ventricular tachycardia

| **Supplementary Table 4. Baseline characteristics of the study population according to whether an ablation procedure was performed.** | | | | |
| --- | --- | --- | --- | --- |
|  | Overall population (n = 85) | Ablation performed (n = 32) | Ablation not performed (n = 53) | p value |
| Age, years, median (IQR)  Male sex, n (%)  BMI, kg/m², median (IQR)  Body surface area, m², median (IQR)  Cardiovascular risk factors, n (%)  Current smoking  Dyslipidaemia  Hypertension  Diabetes mellitus  History of cardiomyopathy, n (%)  Ischemic  Non-ischemic dilated cardiomyopathy  Cardiac sarcoidosis  Arrhythmogenic  Hypertrophic  Congenital  Unknown  Time from cardiomyopathy diagnosis to ES, n (%)  < 6 months  6 months – 5 years  > 5 years  Latest known LVEF, %, median (IQR)  Usual treatment prior to ES, n (%)  Betablockers  Amiodarone  ACEI/ARB  Sacubitril/valsartan  MRA  Loop diuretics  Anticoagulant  Antiplatelet agents  Statin  Ventricular arrythmias, n (%)  Ventricular tachycardia  Ventricular fibrillation  Electrical storm  VT ablation  ICD, n (%)  Secondary prevention  Primary prevention  Single chamber  Dual chamber  Resynchronization therapy | 56.0 (48.0 – 61.0)  73 (85.9)  26.4 (23.5 – 29.2) (n = 83)  1.99 (1.86 – 2.12) (n = 83)  26 (30.6)  28 (32.9)  30 (35.3)  15 (17.6)  78 (91.8)  20 (25.6)  43 (55.1)  1 (1.3)  6 (7.7)  5 (6.4)  1 (1.3)  2 (2.6)  5 (6.4)  11 (14.1)  62 (79.5)  27.0 (20.0 – 35.0)  65 (77.4) (n = 84)  38 (45.2) (n = 84)  41 (49.4) (n = 83)  21 (25.3) (n = 83)  40 (48.2) (n = 83)  53 (63.9) (n = 83)  41 (48.8) (n = 84)  24 (28.9) (n = 83)  29 (34.9) (n = 83)    53 (62.4)  10 (11.8)  27 (31.8)  28 (32.9)  70 (82.4)  38 (54.3)  32 (45.7)  16 (22.9)  19 (27.1)  25 (35.7) | 57.5 (49.3 – 62.0)  27 (84.4)  26.5 (23.3 – 28.8) (n = 31)  1.95 (1.87 – 2.10) (n = 31)  7 (21.9)  10 (31.3)  9 (28.1)  5 (15.6)  26 (81.3)  7 (26.9)  15 (57.7)  1 (3.8)  3 (11.5)  0 (0.0)  0 (0.0)  0 (0.0)  0  5 (19.2)  21 (80.8)  30.0 (20.5 – 36.5)  25 (78.1)  12 (37.5)  14 (45.2) (n = 31)  8 (25.8) (n = 31)  15 (48.4) (n = 31)  18 (58.1) (n = 31)  15 (46.9)  7 (22.6) (n = 31)  9 (29.0) (n = 31)  20 (62.5)  3 (9.4)  10 (31.3)  13 (40.6)  26 (81.3)  15 (57.7)  11 (42.3)  6 (23.1)  7 (26.9)  9 (34.6) | 55.0 (48.0 – 59.0)  46 (86.8)  26.3 (23.5 – 29.6) (n = 52)  2.00 (1.85 – 2.14) (n = 52)  19 (35.8)  18 (34.0)  21 (39.6)  10 (18.9)  52 (98.1)  13 (25.0)  28 (53.8)  0 (0.0)  3 (5.8)  5 (9.6)  1 (1.9)  2 (3.8)  5 (9.6)  6 (11.5)  41 (78.8)  25.5 (20.0 – 30.0)  40 (76.9) (n = 52)  26 (50.0) (n = 52)  27 (51.9) (n = 52)  13 (25.0) (n = 52)  25 (48.1) (n = 52)  35 (67.3) (n = 52)  26 (50.0) (n = 52)  17 (32.7) (n = 52)  20 (38.5) (n = 52)  33 (62.3)  7 (13.2)  17 (32.1)  15 (28.3)  44 (83.0)  23 (52.3)  21 (47.7)  10 (22.7)  12 (27.3)  16 (36.4) | 0.36  0.76  0.74  0.92  0.23  1.00  0.35  0.78  0.35  0.19  0.53  1.00  0.27  0.65  1.00  1.00  0.48  0.83  0.45  0.48  1.00  0.74  1.00  0.34  1.00 |

ACEI/ARB, angiotensin-converting enzyme inhibitor/angiotensin receptor blocker; BMI, body mass index; IQR, interquartile range; MRA, mineralocorticoid receptor antagonist

| **Supplementary Table 5. Clinical, echocardiographic, and biological findings at baseline according to whether an ablation procedure was performed.** | | | | |
| --- | --- | --- | --- | --- |
|  | Overall population (n = 85) | Ablation performed (n = 32) | Ablation not performed (n = 53) | p value |
| Cardiogenic shock, n (%)  Blood tests at admission, median (IQR)  Sodium, mmol/L  Creatinine, µmol/L  Bilirubin, mg/L  Arterial blood lactates, mmol/L  LVEF, %, median (IQR) | 49 (57.6)  137.0 (134.0 – 140.0) (n = 73)  105.0 (87.8 – 136.0) (n = 76)  13.5 (8.2 – 20.0) (n = 62)  1.3 (1.0 – 1.8) (n = 48)  20.0 (15.0 – 30.0) | 14 (43.8)  138.0 (137.0 – 140.0) (n = 31)  98.0 (84.5 – 128.5) (n = 31)  11.7 (7.9 – 15.0) (n = 28)  1.3 (0.9 – 1.8) (n = 21)  22.5 (20.0 – 35.0) | 35 (66.0)  136.0 (132.3 – 139.0) (n = 42)  110.0 (91.0 – 142.0) (n = 45)  16.0 (10.0 – 28.0) (n = 34)  1.2 (1.0 – 1.9) (n = 27)  20.0 (15.0 – 25.0) | 0.07  0.04  0.32  < 0.01  0.62  0.05 |

IQR, interquartile range, LVEF, left ventricular ejection fraction.

| **Supplementary Table 6. Electrical storm characteristics and management in the overall population according to whether an ablation procedure was performed.** | | | | |
| --- | --- | --- | --- | --- |
|  | Overall population (n = 85) | Ablation performed (n = 32) | Ablation not performed (n = 53) | p value |
| Initial reason for hospitalization, n (%)  Ventricular arrythmia (VT, VF, ES, syncope, ICD shock)  STEMI  NSTEMI  Cardiogenic shock, heart failure  Trigger factor, n (%)  STEMI  NSTEMI  Hypokalaemia  Infection  Hyperthyroidism  VT ablation  None  Anti-arrhythmic drugs, n (%)  Betablocker  Amiodarone  Lidocaine  Magnesium sulfate  Deep sedation, n (%)  Stellate ganglion blockade, n (%)  Vasoactive and inotrope agents, n (%)  Dobutamine  Norepinephrine  Epinephrine  Acute mechanical circulatory support, n (%)  ECMO  IABP | 67 (78.8)  5 (5.9)  0 (0.0)  13 (15.3)  7 (8.2)  3 (3.5)  4 (4.7)  2 (2.4)  2 (2.4)  1 (1.2)  66 (77.6)  54 (64.3) (n = 84)  75 (89.3) (n = 84)  37 (44.0) (n = 84)  42 (50.0) (n = 84)  39 (45.9)  5 (5.9)  39 (45.9)  18 (21.2)  5 (5.9)  35 (41.2)  28 (80.0)  7 (20.0) | 28 (87.5)  3 (9.4)  0 (0.0)  1 (3.1)  4 (12.5)  1 (3.1)  0 (0.0)  0 (0.0)  1 (3.1)  26 (81.3)  0 (0.0)  24 (75.0)  25 (78.1)  18 (56.3)  16 (50.0)  18 (56.3)  4 (12.5)  15 (46.9)  6 (18.8)  3 (9.4)  10 (31.3)  9 (90.0)  1 (10.0) | 39 (73.6)  2 (3.8)  0 (0.0)  12 (22.6)  3 (5.7)  2 (3.8)  4 (7.5)  2 (3.8)  1 (1.9)  40 (75.5)  1 (1.9)  30 (57.7) (n = 52)  50 (96.2) (n = 52)  19 (36.5) (n = 52)  26 (50.0) (n = 52)  21 (39.6)  1 (1.9)  24 (45.3)  12 (22.6)  2 (3.8)  25 (47.2)  19 (76.0)  6 (24.0) | 0.02  0.52  0.16  0.02  0.11  1.00  0.18  0.06  1.00  0.79  0.36  0.18 |

ECMO, extracorporeal membrane oxygenation; ES, electrical storm; IABP, intra-aortic balloon pump; NSTEMI, Non-ST-segment elevation myocardial infarction; STEMI, ST-segment elevation myocardial infarction; VF = ventricular fibrillation; VT = ventricular tachycardia
